# Using population viability analysis to evaluate management activities for an endangered Hawaiian endemic, the Puaiohi (*Myadestes palmeri*)

**DOI:** 10.1371/journal.pone.0198952

**Published:** 2018-06-13

**Authors:** Jean Fantle-Lepczyk, Andrew Taylor, David C. Duffy, Lisa H. Crampton, Sheila Conant

**Affiliations:** 1 Department of Biology, University of Hawai‘i at Mānoa, Honolulu, HI, United States of America; 2 Pacific Cooperative Studies Unit, Department of Botany, University of Hawai‘i at Mānoa, Honolulu, HI, United States of America; 3 Kaua‘i Forest Birds Recovery Project, Hawai‘i Division of Forestry and Wildlife and University of Hawai‘i at Mānoa, Hanapepe, HI, United States of America; Sichuan University, CHINA

## Abstract

Evolution in the Hawaiian Islands has produced a unique avian assemblage. Unfortunately, many of these bird species are highly endangered or extinct. Despite numerous and increasing threats and great effort aimed at saving endemic birds, we lack basic science necessary for understanding many species of concern. One such species is the critically endangered Puaiohi (*Myadestes palmeri*), a rare songbird endemic to the island of Kaua‘i and the only remaining native thrush on the island. At present, the Puaiohi’s breeding population is estimated to be ~500 birds restricted to the Alaka‘i Wilderness Preserve. We collected demographic data from 2007–2012 and supplemented it with published sources. Using Vortex, we developed stochastic population models to represent Puaiohi population dynamics under current and potential management scenarios to determine management’s potential efficacy in aiding species recovery. Management scenarios modeled included rat control, habitat improvement, general survival facilitation, and provision of nest boxes. The model indicated a decline in abundance with a growth rate (r) of -0.267 under baseline conditions. Female and juvenile survival appeared to be the most influential parameters related to population growth and persistence, so management should focus on increasing female and juvenile Puaiohi survival. Rat control, even at more conservative levels, appeared to be the most effective method of increasing Puaiohi abundance. Our results indicate that practical, attainable management activities can increase Puaiohi and bring the species back from the brink of extinction. Such findings provide an example for other endangered species conservation efforts.

## Introduction

The Hawaiian Islands are home to a rare and evolutionarily unique, but rapidly disappearing, assemblage of birds [1, 2]. Once home to 152 land bird species, 110 of these have gone extinct since human arrival [3]. Thirty-three of its remaining 42 endemic birds are listed as endangered or threatened, making Hawai‘i home to one of the most endangered avifaunas in the world [3,4]. However, despite these great threats and relatively large management expenditures directed at saving endemic birds, some of the most basic science necessary for understanding species of concern has not been done [5]. For instance, population models and population viability analyses (PVA) have not been conducted for 66% of Hawaii’s endangered avifauna, representing a critical gap in knowledge. This lack of knowledge is problematic, given that population viability is a criterion used for down listing (or de-listing) Puaiohi and its recovery plan lists population modeling as a recovery objective for the species [6]. To accurately assess the viability of these endangered species in the face of a changing climate, invasive species, and human population growth, and to provide the basis for conservation of these critical species, population models and PVA are urgently needed.

The Endangered Puaiohi, or Small Kaua‘i Thrush, (*Myadestes palmeri*) is one of the critically endangered [7] bird species that has yet to be evaluated in terms of its population dynamics. Endemic to Kaua‘i, the Puaiohi is the only remaining avian native frugivore on the island so it may play a critical role in the persistence of native plant species and their associated invertebrates. The Puaiohi is also one of the last six endemic forest bird species to remain in Kauai’s Alaka‘i Swamp [8]. Although some of these species were rare at the turn of the 20^th^ century [9], most were more common than the Puaiohi. In fact, its larger congener, the Kāma‘o, was once the most common forest bird on Kaua‘i [9, 10], yet the Puaiohi has persisted while the Kāma‘o is now extinct.

The Puaiohi has experienced range contraction since the 1960s [6, 10], as it is no longer found at lower elevations (1,000–1,050 meters) and is currently restricted to a remnant of the Alaka‘i Wilderness Preserve at 1,050 to 1,300 meters. Over the past 20 years, three different estimates of Puaiohi population have been calculated, using different techniques in terms of approach and rigor [11]. While these estimates have overlapping ranges in population size (300–500 birds using data from 1995–1998, 270–525 birds using data from 2003–2005, 414–580 birds using data from 2011–2013), generating an assumption of population stability, data types and methods varied, complicating comparisons. Given the lack of consistent approaches, long-term population trends remain unclear. Several factors are thought to affect population vulnerability, including drought, hurricanes, mammalian (rat [*Rattus* spp.] and cats [*Felis catus*]) predation at all life stages, disease (particularly infection with avian malaria [*Plasmodium relictum*] and potentially avian pox [*Avipoxvirus* spp.]), and habitat degradation due to feral livestock (pigs and goats) and invasive plants [6, 12, 13, 14, 15]. The Puaiohi’s preference for nesting along stream banks on fern-covered ledges may limit the availability of suitable nest sites, particularly because invasive plants often cover cliff faces [14].

Given the Puaiohi’s small population size and range, it is essential to gain a better understanding of the conditions which affect its recruitment and survival, as it faces many potential hazards of unknown impact. One useful tool conservation biologists can use to quantify the risk of extinction and to examine the relative benefits of alternative management actions is population viability analysis (PVA) [16] which incorporates demographic and environmental variables to forecast population persistence and extinction risk. Although the significance of the actual quantitative model results may be somewhat limited, PVA is useful for testing the relative importance of model parameters via sensitivity analysis, evaluating management strategies, and identifying priorities for maximizing effective species recovery [17, 18, 19, 20]. Such model revelations provide conservation biologists with insights into where they need to devote resources in order to develop the best possible parameter estimates and which demographic characteristics of the population are the most efficacious to manage in terms of conserving the population. In addition to providing information on specific demographic factors, PVA can be used to rank management options amongst a suite of possible activities (e.g., [21, 22, 23]). This predictive power allows managers to explore the possible outcomes of a suite of management options without risking potentially ineffectual ones on a species that needs immediate help.

The goal of this research is to inform Puaiohi conservation by comparing potential management options, and to use these comparisons to develop best management practices. To address this goal, we modeled Puaiohi populations under current and potential future management scenarios to determine their potential efficacy in aiding in the recovery of this rare and ecologically important species. We predicted that extinction probability would decrease and population growth rate increase if predators were controlled, safer nesting alternatives and/or supplemental food were provided, or survival was increased via control of malaria or other methods. Because many of the issues facing Puaiohi are the same as those faced by the other Hawaiian forest birds, this research may therefore provide a template for similar approaches for other forest birds of Hawai‘i.

## Methods

### Study area

The primary data used for calculating parameters were collected from 2005–2011 at four sites in the Alaka‘i Wilderness Perserve (22.0897° N, 159.5619° W): Kawaikōī, Koaie, Mohihi, and Halepa‘akai. All data used in this paper were collected by the Kaua‘i Forest Bird Recovery Project, a cooperative project of the Pacific Cooperative Studies Unit of the University of Hawai‘i and the State of Hawai‘i Department of Land and Natural Resources, Division of Forestry and Wildlife. The Hawai‘i Division of Forestry and Wildlife permitted access to the Nā Pali Forest Reserve and the Alaka‘i Wilderness Preserve, while the Hawai‘i Division of Forestry and Wildlife and the U.S. Fish and Wildlife Service provided permits to work with Puaiohi. The study sites ranged in elevation from 1,123–1,303 meters above sea level and occurred in the native wet and mesic forests in the Alaka‘i Plateau inhabited by Puaiohi. These forests are dominated by ‘ōhi‘a (*Metrosideros polymorpha*), koa (*Acacia koa*), ōlapa (*Cheirodendron trigynum*), lapalapa (*C*. *platyphyllum*), ‘ōhi‘a ha (*Syzygium sandwicensis*), kāwa‘u (*Ilex anomala*), and kōlea (*Myrsine lessertiana*), with a diverse understory of native plants including ‘ōhelo (*Vaccinium calycinum*), and kanawao (*Broussaisia arguta*) [6]. These forests are amongst the wettest in the world, with annual rainfall averaging 6.5 m [24].

### PVA software

We conducted our PVAs using Vortex 10 [25] which simulates stochastic demographic and environmental processes. Vortex is an individual-based simulation model that follows the fates of each animal in the simulated population from birth to death, with all events happening according to defined probabilities.

### Baseline model inputs

Within Vortex (Table 1), we developed baseline models using all available information on Puaiohi. Each model was simulated 1,000 times [26], over a time frame of 25 years. Although long term preservation of the species is the ultimate goal, we felt the recovery program is currently driven by urgent short-term needs. Furthermore, longer term time spans tend to produce higher extinction probabilities [27], propagate errors [17], and produce more uncertain events [28]. Thus, we chose the relatively short time frame of 25 years because it allowed for exploring and testing the immediate effects of management strategies while minimizing the effects of uncertainties or errors in our parameter estimates. We defined extinction as occurring when only one sex remained.

**Table 1:** Vortex parameter inputs for the baseline Puaiohi population model.

Parameter	Value
**Species Description**	
Inbreeding Depression	
Lethal Equivalent	6.29
% due to Recessive Lethals	50%
EV Concordance of Repro and Survival?	Yes
**Reproductive System**	
Reproductive System	monogamous, probably long term
Age of 1st Offspring Females	1
Age of 1st Offspring Males	1
Max Age of Repro	10
Max # Broods/Year	4
Max # Progeny/Brood	2
Sex Ratio at birth in % Males	1 to 1
Density Dependent Reproduction	yes
% Breeding at Low Density	100%
% Breeding at K	90%
Allee Parameter	0
Steepness Parameter	8
**Reproductive Rates**	
% Adult Females Breeding	Will be automatically calc'd from % Breedings, A, and B
EV (SD) in % Breeding	10
Distribution of Broods each Year	0–13.16%; 1–42.11%; 2–36.84%; 3–5.26%; 4–2.63%
# Offspring/Female/brood (exact distribution of brood size)	1–30.77%; 2–69.23%
**Mortality Rates**	
Mortality of Females as %	
Mort from 0 to 1	0.77
SD in Mort from 0 to 1	10
Annual Mort after Age 1	0.54
SD in Mort after Age 1	3
Mortality of Males as %	
Mort from 0 to 1	0.77
SD in Mort from 0 to 1	10
Annual Mort after Age 1	0.29
SD in Mort after Age 1	3
**Mate Monopolization**	
% Males in Breeding Pool	100%
**Initial Population Size**	
Stable Age Distribution?	Yes
Initial Population Size	500
**Carrying Capacity**	
K	1100
SD in K due to EV	10

Four cohorts or stages were modeled: Juvenile females, adult females, juvenile males, and adult males. We developed our demographic input parameters from a variety of sources, including original data, previously published information, and discussion with experts on the species. Because little is known about the genetics, effects or impacts of inbreeding depression on Puaiohi, we developed models both with and without inbreeding depression included (Table 2); for the model incorporating inbreeding, we used the default heterosis model of 6.29 lethal equivalents, 50% due to recessive lethals. We tested whether inclusion of inbreeding depression mattered using equivalency testing in Minitab 17.3.1. Equivalence testing is a statistical tool used to test whether observations from two groups are similar enough to be biologically analogous. In equivalence testing, the null hypothesis is that the difference between the means is greater than a researcher-defined amount, which is referred to as “interval of tolerable difference.” We defined the limits at which we considered model output differences to be equivalent as ± 0.02 stochastic r (essentially ± 2% annual growth rate) and ± 10 individuals remaining at 25 years. We found inbreeding depression models to be equivalent to non-inbreeding models based upon these defined limits (p < 0.021), and therefore did not include the variable in the model. We assumed that the effects of environmental variation on reproduction and survival were correlated.

**Table 2:** Baseline candidate model results. The model in bold, Baseline without inbreeding, was chosen as the baseline for all model scenario comparisons. Equivalency testing of model output (stochastic r and N extant) shows models with and without inbreeding depression to be equivalent, so we chose to exclude inbreeding depression. The Baseline without inbreeding model, with a steepness value of 8, was equivalent to models with steepness 4 or steepness 16. As a result, we chose to use a steepness value of 8 in our baseline model.

Model	Prob of Extinct	PE SE	Stoch r mean	Stoch r SE	Stoch r SD	N in all pops mean	N SE	N SD	TE median	TE mean	TE SE	TE SD
Baseline w. inbreeding	0.981	0.004	-0.269	0.002	0.196	0.20	0.02	0.75	17.00	16.64	0.11	3.37
**Baseline wo. inbreeding**	**0.974**	**0.005**	**-0.267**	**0.002**	**0.201**	**0.32**	**0.04**	**1.24**	**17.00**	**16.72**	**0.11**	**3.53**
Baseline wo. inbreeding, steep4	0.966	0.006	-0.264	0.002	0.199	0.35	0.03	1.07	17.00	16.92	0.11	3.48
Baseline wo. inbreeding, steep16	0.973	0.005	-0.268	0.002	0.199	0.28	0.03	1.06	17.00	16.69	0.11	3.52

#### Reproduction

We assumed Puaiohi are monogamous [15], and pairs probably persist from year to year, provided mates survive. Individuals of both sexes can breed at a year old. Though one study observed after hatch year birds helping at 8% of nests, suggesting they may have needed to delay reproduction due to some sort of limiting factor [29], helping behavior has not been seen in other studies. Thus, we assumed that, in general, birds would breed at one-year old, and would breed each year. Puaiohi always lay two eggs per clutch [14], and during the 2007–2009 study period fledged one chick in 30.77% of attempts and two chicks in 69.23% of attempts. Previous studies found up to five nest attempts per season [29], while we never saw more than four true nest attempts per season between 2007 and 2009. As a result, we assumed four nest attempts to be a reasonable maximum [during the 2007–2009 study period the distribution of successful nest attempts was: 0 (13.16%), 1 (42.11%), 2 (36.84%), 3 (5.26%), and 4 (2.63%)]. A Puaiohi mist-netted as a hatch-year bird in 1965 survived in captivity until it was 11 years old [29]. The longest lived captive female died at 16 years old at the Maui Bird Conservation Center (MBCC), but stopped laying eggs at nine years old. The oldest captive male lived for 13 years and was still able to fertilize eggs the breeding season before he died and other individuals have lived as long as 10 years (S. Belcher, personal communication). As a result, we decided that a maximum life span of 10 years was reasonable. Of 118 eggs laid at the MBCC, 57 hatched males and 61 females (S. Belcher, personal communication), which we used as the basis for a 1:1 sex ratio at hatch.

#### Density dependence

Vortex models density dependence in terms of its effect on reproduction as: P(N) = (P(0)-((P(0)-P(K))*((N/K)^B)))*(N/(A+N)); where P(0) is the percentage of adult females breeding at low density; P(K) is the percentage of adult females breeding at carrying capacity; N is initial population size; K is carrying capacity; B is a steepness parameter, which determines the shape of the curve relating the percentage of adult females breeding to population size; and A is the Allee parameter, which accounts for the decrease in the proportion of females breeding at low densities, due to of the increased difficulty of finding a mate at low population densities. We assumed that Puaiohi would be density-dependent, given that it is a territorial bird with limited nesting sites and food sources. During intensive territory mapping from 2007–2009, an average of 90% of the population encountered was breeding. However, by the 2009 census, fecundity appeared to drop off slightly, perhaps due to the population nearing K. Thus, we assumed 90% of the population was breeding at K. Given that a large proportion of the population still seemed to be breeding at K, we assumed that 100% of the population would breed at low density. In the absence of data to the contrary and given that Puaiohi occur in a relatively small remnant habitat area we assumed the Allee effect was zero. To determine the appropriate steepness parameter, we graphed a series of density dependent population projection plots using steepness parameters from 0.25 to 16. A steepness parameter of 8 looked the most reasonable, although 4 and 16 also seemed plausible. To test the influence of the steepness parameter, we ran three versions of the baseline without inbreeding model, with steepness values of 4, 8, and 16 (Table 2), and as with inbreeding, tested for equivalency with Minitab 17.3.1, within the self-defined limits of ± 0.02 stochastic r and ± 10 individuals remaining at 25 years. Because all models showed equivalent results (p < 0.03), we chose to use a steepness value of 8 in our baseline model. Vortex used the parameters of percent breeding at low density and percent breeding at high density, the Allee parameter and the steepness parameter to automatically calculate the percent of adult females breeding. For the environmental variation in percent breeding, we used default values of 10% in the absence of other data. We do know that except in extreme weather years, fecundity rates, at least, are fairly stable [30]. Finally, we assumed that 100% of males would be in the breeding pool, though all may not find mates due to limited females resulting from higher female mortality [31].

#### Mortality

Using mark-recapture analysis to estimate the annual survival of juvenile and adult Puaiohi, previous research found that adult males survived at a higher rate (71 ± 9% SE) than females (46 ± 12% SE), and indicated that rat predation may be a significant factor in female mortality [31], a pattern common in other Hawaiian passerines [32, 33,34,35]. Additionally, previous research found juvenile survival to be quite low (26 ± 21% SE) [31], which may limit population growth. Recently fledged young tend to remain on the ground for about four days after fledging, making them particularly vulnerable to predation by rats and feral cats. We used these survival estimates in our baseline model, as they represent the only long-term estimate of Puaiohi survival rates. Given the relatively high mortalities of juveniles and females and the low starting population, we found our model declined rapidly to zero with environmental variations in mortality rate greater than 10%. Furthermore, as with percent breeding, we assumed that in this relatively stable island environment we would not see large annual fluctuations in mortality rates. Thus, we used Vortex’s default, relatively modest deviation in mortality rate of 10% for juvenile mortality and 3% for adult mortalities.

#### Population size and carrying capacity

In the absence of information to the contrary, we assumed Puaiohi achieve a stable age distribution. Estimates of population size have varied, from conservatively exceeding 200 [29] to around 500 [6, 11]. We used the more recent USFWS estimates in our models as they were the most recent and based on the longest-term dataset. No attempts have been made to estimate Puaiohi carrying capacity in the Alaka‘i Swamp. However, the Puaiohi population is strongly linked to streams, and there have been estimates of bird densities along those streams. Specifically, within Puaiohi habitat, there are ~221 km of streams, about half of which had enough cliff face to be suitable for nesting. At peak nesting density in 2009, with nest success declining (perhaps indicating the population was approaching K), we found 27 nesting territories over 6 km of suitable habitat. Assuming two Puaiohi per territory, this yields an upper limit of 1,062 birds over the 118 km of suitable habitat. For our estimate of K, we rounded up to 1,100 to include in the population any unpaired or floater birds also utilizing the resources. This may be an overestimate of K, as these numbers are estimated from one of the higher nest density streams, and not all streams are likely to support this number of birds. However, in the interest of testing increases in other demographics of interest, we choose to err on the side of overestimation to give our modeled populations some room for increase. In the absence of better information, we assumed fluctuation in K due to environmental variation to be ±10%.

#### Software output

Vortex’s standard output provided us with stochastic r, probability of extinction within 25 years, average population size at 25 years, median time to extinction (provided 50% of simulations went extinct), and mean time to extinction for populations that went extinct within 25 years.

### Sensitivity analysis

We performed sensitivity analysis to understand how uncertainty about parameter values affect model outcomes [36]. We varied each key parameter by ±10%, ±25%, and ±50%, while holding all other parameters to baseline values. The parameters examined were starting population, carrying capacity, and juvenile, male, female and combined adult mortalities, number of reproductive attempts per year, and number of young produced per attempt. We used a standard sensitivity index (S_*x*_) [37] for sensitivity analysis. Baseline model values were used to compute the standard sensitivity index for each parameter, which was calculated as:

$$S_{x}=\left(x_{new}-x_{baseline}\right)/\left(P_{new}-P_{baseline}\right)$$ Eq 1

where x is the output value (stochastic r/λ or N extant) and P is the parameter of interest.

Since the model’s sensitivity to mortality at all life stages was of particular interest to us, we also wished to evaluate what levels of error in mortality estimates would provide a stable population projection. To investigate this further, we decreased all mortality measures by 10%, 15%, 17.5%, 20%, 25% in order to determine what magnitude of decrease would be necessary to produce r = 0.

### Management models

After assessing the relative influence of each individual input parameter, we used the best available data to develop a suite of potential management scenarios. The four management scenarios developed included rat management, habitat improvement, survival assistance, and the provision of nest boxes (Table 3). While these scenarios do not cover all threats faced by the species, they address the primary threats and limiting factors identified in the species recovery plan [6]. To reflect the uncertainty in some of our estimations, we developed two levels of each management activity—standard and conservative.

**Table 3:** Vortex parameter input for management models.

Model	K	Juv mort	Female mort	Male mortality	Fecundity (successful attempts/ year)	Fecundity (fledged/ successful attempt)
Baseline	1100	0.77	0.54	0.29	1.42	1--30.77%; 2--69.23%
Rat control	1375	0.58	0.29	0.29	2.13	1--30.77%; 2--69.23%
Rat control-conservative	1210	0.69	0.41	0.29	1.78	1--30.77%; 2--69.23%
Nest boxes	1375	0.77	0.29	0.29	2.13	1--30.77%; 2--69.23%
Nest boxes-conservative	1210	0.77	0.41	0.29	1.78	1--30.77%; 2--69.23%
Survival assistance	1100	0.58	0.41	0.22	1.42	1--30.77%; 2--69.23%
Survival assistance-conservative	1100	0.69	0.49	0.26	1.42	1--30.77%; 2--69.23%
Habitat improvement	1210	0.69	0.49	0.26	1.95	1--13.46%; 2--86.54%
Habitat improvement-conservative	1210	0.69	0.49	0.26	1.56	1--23.85%; 2--76.15%

For the optimistic rat management models, we decreased female mortality to that of males, since recent work [31] suggests than mortality due to nest predation by rats is the leading driver behind the higher mortality rates in females. We surmised that in a best-case scenario, removal of rats would produce female mortality rates akin to males. In the O‘ahu ‘Elepaio, rat control has been shown to increase female survival by 10%-27% [33, 34] which may make our optimistic estimate slightly high. We increased the number of successful attempts/year by 50%, as previous research [14] found that 50% more nests fledged young with rat control. No experimental data exists to quantify rat predation effect on juvenile mortality. However, we surmised that rat removal would have less impact on juvenile mortality than on adult female mortality, as juveniles are probably subject to a greater range of mortality effects, and they are likely mostly vulnerable to rat predation in just the first days after fledging. As a result, we decreased juvenile mortality by 25%. We presumed the removal of rats would not have much effect on male mortality, since males do not incubate eggs on the nest (when females are presumably most vulnerable to rat predation) nor would rat removal affect the number of young fledged/attempt since if a rat found a nest, it would likely kill both chicks, resulting in a failed attempt. Since rats are known to consume a variety of native fruits and may damage native fruiting plants [1, 10], we assumed that they may have some effect on carrying capacity of Puaiohi as competitors for preferred food sources. As a result, we increased carrying capacity by 25%.

In our conservative rat management model, we decreased female mortality by only 25% to allow for incomplete rat control as well as the possibility of other causes of female mortality during breeding. We lowered the increase in number of successful attempts per year to 25% more than baseline to allow for any overestimates in a previous experiment [14], and for other causes of nest failure. Finally, in the absence of data, in our more conservative model we lowered juvenile mortality by 10% and increased carrying capacity by 10%. Again, we assumed rat removal would not affect male mortality or the number fledged per attempt.

In our nest box models, we assumed that we can eventually design a rat proof nest box design that will be readily used by Puaiohi. In this event, females and nests would be protected from rat predation, but there would be no effect on juvenile survival as they would have not added protection once they left the nest. Furthermore, assuming nest sites are a limiting factor, providing more nest sites would increase carrying capacity. As a result, in our optimistic nest box model, we decreased female mortality to that of males, and increased the number of successful attempts/year by 50%, as was done in the rat management models. We also increased carrying capacity by 25%. In our conservative nest box models, we decreased female mortality by 25%, increased the number of successful attempts per year by 25%, and increased K by 10%. We assumed no effect on male mortality, the number of young fledged per attempt, or juvenile mortality.

In our survival assistance models, we explored the effect of just increasing Puaiohi survival at all post-fledging life stages, with no increases in fecundity or other factors. While Puaiohi mortality has likely increased due to several factors of recent origin, including the predatory issues explored in the previous two models, malarial infection is also a concern, though the extent of its impact on the population remains unclear. Avian malaria (*Plasmodium relictum*) was detected in 22.7% of birds tested in 2007–2008 [38]. This high prevalence may indicate that at least some Puaiohi survive acute infection and have some tolerance of malaria [39]. There is evidence, albeit based on relatively small sample sizes, that survivorship of Puaiohi with chronic malaria is as good as those without malaria [31], but presumably some birds succumb to their first bout of malaria. Given the Puaiohi’s absence from the lower elevation, where malaria is more prevalent, it seems likely that malaria does have some impact on the population, though habitat degradation and weed prevalence likely influences this distributional pattern as well. To attempt to model how eliminating the effects any non-predator related mortality factors, including malaria, might impact the Puaiohi population in the absence of any other population effects, we decreased mortality at all life stages (juvenile, adult female, and adult male) by an optimistic 25%. In the more conservative survival assistance model, we decreased all mortality by 10%. In the specific case of malaria, there is some evidence of that infection can cause nestling mortality, thereby reducing fecundity [40]. However, in the interest of keeping these models more general and to differentiate them from the habitat models which follow, we have not incorporated these effects in the survival assistance models.

Our final models looked at potential impacts of habitat improvements on the Puaiohi. This could be as the result of supplemental feeding of fruits as was provided to released, captive bred Puaiohi. Alternatively, habitat could be improved by controlling invasive species that outcompete native food sources, potentially decreasing food availability, particularly during the breeding season. Previous research by Snetsinger et al. [14] saw a 37.5% increase in nest attempts per year in wet years over drier years, presumably due at least in part to increased food supply following higher rainfall. Snetsinger et al. further noted a 54.5% increase in the number fledged per attempt in the wet year over the mean number fledged over the three years of study. However, one really dry year brings the mean down and likely over estimates this impact. Considering these findings, we increased the number of successful nesting attempts per year by 37.5% and number of young fledged/attempt by 25% (lower than Snetsinger et al.’s finding due to our concern of overestimation) to reflect the greater food supply by supplementation. We also assumed that increased food supply could slightly increase carrying capacity and slightly decrease juvenile, male, and female mortality, so those parameters were adjusted by 10%, accordingly. In our more conservative habitat improvement model, we increased number of attempts per year and young fledged per attempt and carrying capacity by 10% and decreased juvenile, male and female mortality by 10%. In both the optimistic and conservative habitat improvement models, we held all other parameters to baseline values.

To compare between management models, we describe results of stochastic r, mean population size, probability of extinction, and mean and median time to extinction in relation to one another. Specifically, we describe similarities and difference between management models as well as trends. We did not test for statistically significant differences among models because the results would be heavily dependent on our choice of how many replicate iterations of each model to use [41]. As a result, the model outputs are meant to provide guidance on management approaches that are likely to be successful relative to one another.

Recent work on the utility of PVAs to inform management decisions has suggested using the model output more explicitly to account for likelihood of success when a management action is performed compared to no action [42]. Specifically, evaluating the distributions of PVA parameters, such as rate of growth, between a baseline model and a management model allows for determination of success or failure by engaging in a management action. Using the approach of Robinson et al. [42], we evaluated Vortex model output for r between baseline and management models in order to determine what percent of the time the management models would lead to one of three outcomes: success, failure, or management not needed, as well as the probability that management worked when needed (calculated as Success/Success + Fail). From the raw data output by Vortex, we calculated the stochastic intrinsic growth rate (stochastic r) for each population iteration within a management scenario. In instances where the iteration experienced extinction before the end of the 25 years, we used time to extinction as *t*. If the number of individuals in the iteration hit carrying capacity (K) and oscillated around it, we truncated the time period to just before the population hit K (e.g., *t* = time to first year it exceeds 1,000, which is just below K, as density dependence started to influence the population’s growth rate). If a population iteration failed to hit K, or hit K once and then declined, but did not go extinct by end of 25 years, then we used a *t* of 25.

## Results

**Fig 1:**
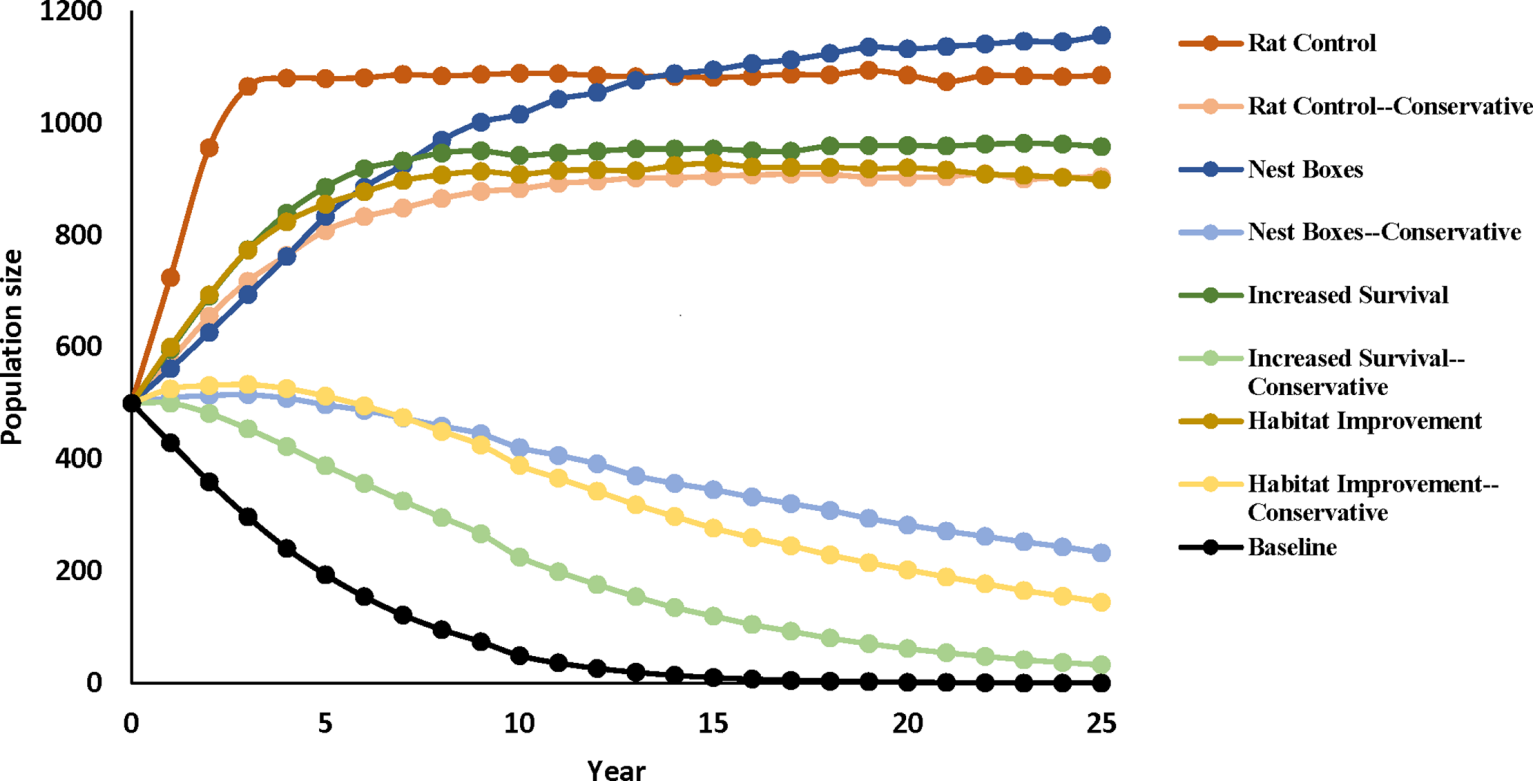
Population trajectories of baseline and management models in Vortex.

The baseline model yielded a declining population (r = -0.27; Fig 1, Table 4). Probability of extinction was high (0.97), with nearly all populations going extinct. Furthermore, the mean time to extinction was 16.72 years, while median time to extinction was 17 years with a mean population size of 0.32 birds.

**Table 4:** Population viability model results from Vortex modeling software. Probability of extinction and population size are within 25 years, and mean time to extinction is for all iterations that went extinct within 25 years.

Model	Stoch r	Prob of extinct	Mean N	N SD	Median TE	Mean TE	TE SD
Baseline	-0.267	0.97	0.32	1.24	17	16.72	3.53
Rat control	0.297	0	1344.15	124.8	0	0	0
Rat control-conservative	0.075	0	972.21	207.36	0	0	0
Nest boxes	0.082	0	1156.68	205.34	0	0	0
Nest boxes-conservative	-0.048	0.01	234.96	221.3	0	23	2.33
Survival assistance	0.075	0	958.02	117.02	0	0	0
Survival assistance-conservative	-0.126	0.16	33.01	38.57	0	22.12	2.46
Habitat improvement	0.059	0	899.18	245.71	0	0	0
Habitat improvement-conservative	-0.067	0.02	144.95	146.54	0	22.8	2.46

### Sensitivity analysis

**Fig 2:**
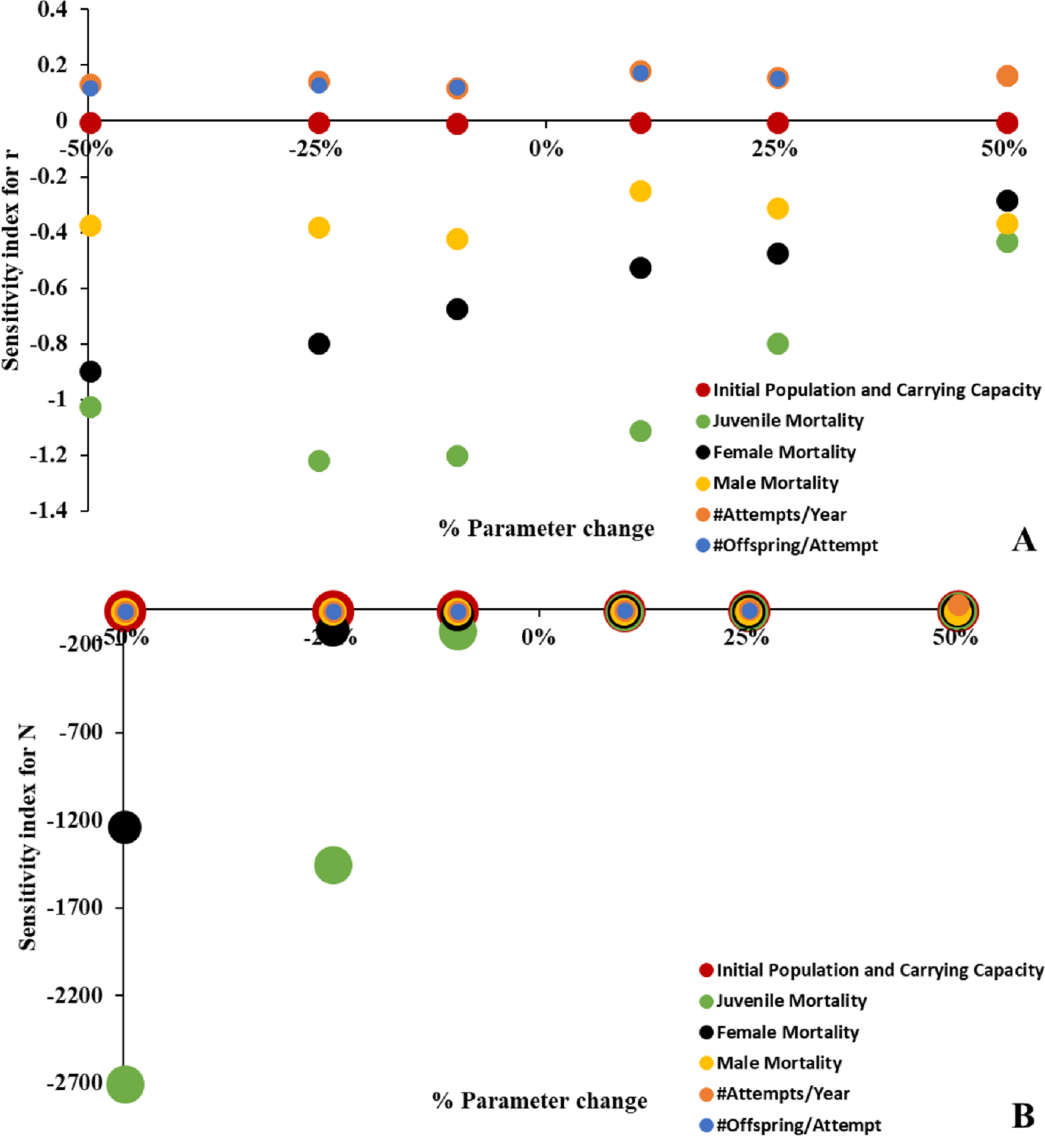
Sensitivity index (SI) of growth rate (A) and final population size (B). Increased distance from X axis (sensitivity index of 0) indicates more sensitivity.

Our models had little sensitivity to either initial population size or carrying capacity (Fig 2). In fact, varying either of these even ±50% had little impact on either growth rate or population size. The model was slightly more sensitive to perturbations in fecundity measures, although not nearly as sensitive as it was to mortality measures (Fig 2). In general, juvenile mortality was the most influential parameter in terms of both growth rate and population size, particularly for changes in the ±25% range. However, both growth rate and N are sensitive to adult mortality, with female mortality being relatively more influential than that of males at all levels of parameter change.

**Fig 3:**
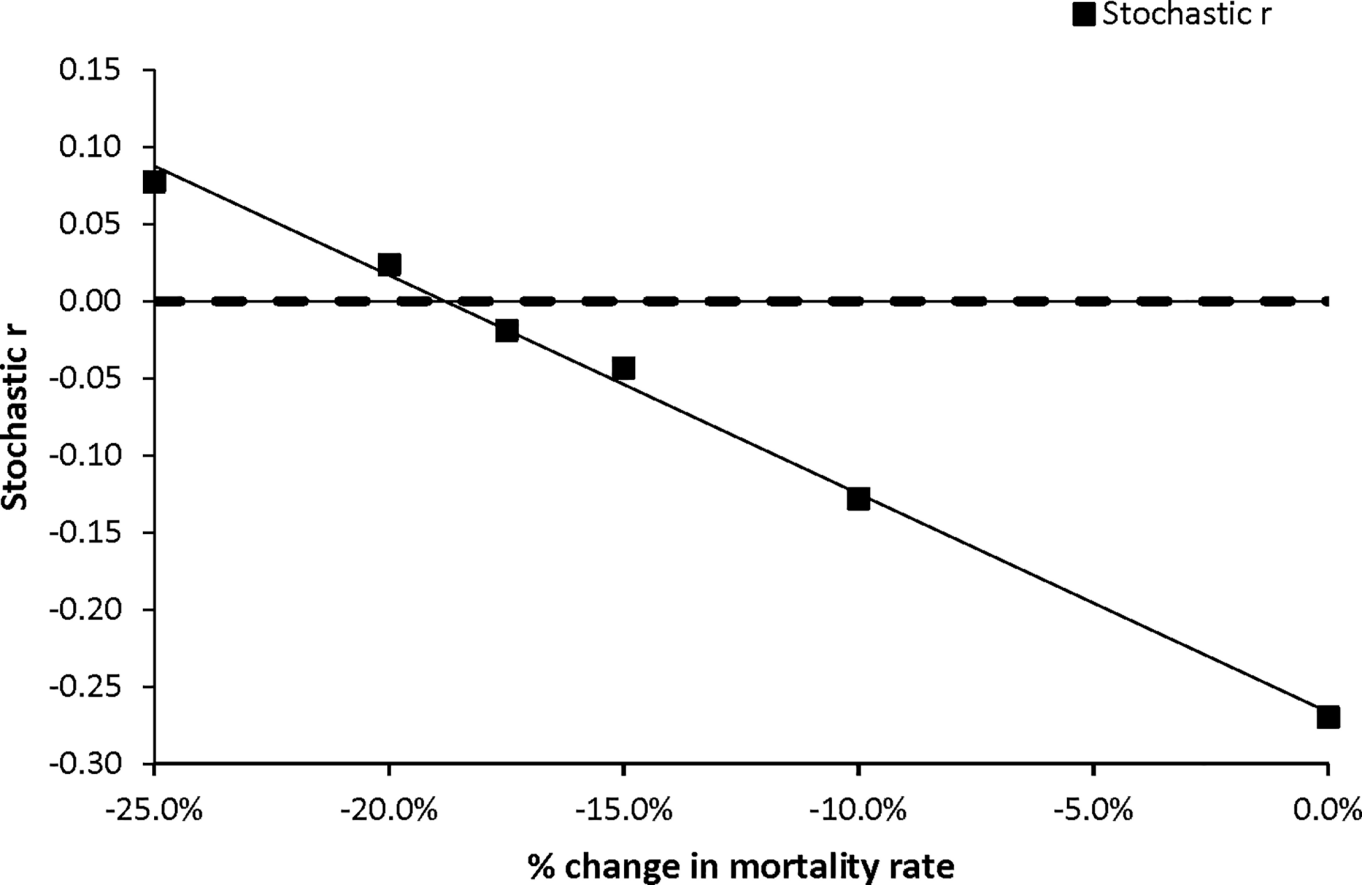
Evaluation of varying mortality rate to achieve a stable growth rate in Vortex.

While our baseline model predicts a rather steeply declining population, scientists have assumed Puaiohi populations have been stable for the last 40 years [6]. Given the models’ sensitivity to mortality, we were concerned that inaccuracies in estimation of mortality may have resulted in the seemingly overly pessimistic predictions of our model. To investigate this further, we decreased all mortality measures by 10%, 15%, 17.5%, 20%, 25%. We found that stable growth (r = 0) was achieved with a decrease in mortality between 17.5% and 20% (Fig 3).

### Management models

Overall the eight management models indicated increases in growth rate, population size, and time to extinction, and lower probabilities of extinction (Table 4) over the baseline model, although these improvements were less pronounced in the conservative versions of the nest box, supplemental feeding and survival assistance models, in which the population still declined fairly rapidly. While our baseline model exhibited negative growth rates, both the conservative and non-conservative rat control, as well as the non-conservative nest box, non-conservative supplemental feeding and non-conservative survival assistance models all exhibited positive growth rates. Although the conservative nest box, conservative supplemental feeding and conservative survival assistance models growth rates remained negative, they were still an improvement over baseline. For probability of extinction, the baseline model had a higher probability of extinction than all other models, with only the conservative supplemental feeding management model showing much risk of extinction. Population size was also markedly larger in management scenarios as compared to the baseline model, even in those scenarios exhibiting a negative growth rate. Finally, of models with iterations that went extinct, there were notable, if modest increases in mean time to extinction between baseline vs. management models. Median times to extinction were not produced for any management models since less than 50% of simulation iterations went extinct for all eight management models.

Of the eight management models, rat control resulted in the largest growth rates, increased population sizes, and zero risk of extinction (Table 4). The nest box model had smaller growth rates, but resulted in a higher population size, again with no risk of extinction. Survival assistance, habitat improvement, and conservative rat control resulted in positive growth rates, increased population size, and longer time to extinction, though of smaller magnitude than rat control and nest box provision models (Fig 1). Conversely, the conservative nest box, conservative survival assistance, and conservative habitat improvement models produced greater times to extinction, growth rates, and final population sizes than baseline models, although they did not result in positive growth rates.

Evaluating the management model outcomes relative to the baseline model showed a variety of responses in terms of either success or failure. Both rat control models demonstrated nearly 100% success in increasing growth rates to at least the target value (Table 5). However, for the other 3 types of management actions, only the standard versions of the models showed great success when compared to their conservative counterparts. Furthermore, even if our baseline is pessimistic and the Puaiohi population is in fact stable, all four standard models and the conservative rat control model still exhibit considerable improvement over current conditions.

**Table 5:** Management model outcomes. Percentages represent the proportion of 1,000 iterations that resulted in one of three possible outcomes (Success, Not Needed, or Failure, see Robinson et al. (2015) for details). Also included is the probability that growth rate rose above stochastic r = 1 when starting below the target (management worked when needed, calculated as *Success*/*Success* + *Fail*).

	Success	Management Not Needed	Failure	Management Worked
Rat control	100%	0%	0%	100%
Rat control-conservative	98.3%	0%	1.7%	98%
Nest box	99.1%	0%	0.9%	99%
Nest box-conservative	11.9%	0%	88.1%	12%
Survival assistance	100%	0%	0%	100%
Survival assistance-conservative	0.2%	0%	99.8%	0%
Habitat improvement	96.6	0%	3.4%	97%
Habitat improvement-conservative	4%	0%	96%	4%

## Discussion

The baseline models indicated negative growth trends and a high probability of extinction in the next 25 years. Because the population has been considered stable historically, it is possible that inaccuracies in parameter estimation may be contributing to our overly pessimistic population predictions. Given the model’s sensitivity to estimates in mortality/survival, and the difficulty in obtaining these estimates, we suggest that this inaccuracy is the most likely cause. In fact, mortality errors of -17.5% to -20% would result in annual juvenile survival of 36%-38%, female survival of 55%-57%, and male survival of 76%-77%. These estimates, which are well within the 95% confidence limits of survival estimates [31] would lead to stable population growth rates. However, even though relatively small errors in survival estimates may affect our baseline models, it should be noted that two other members of the Alaka‘i bird community, the ‘Akeke'e (*Loxops caeruleirostris*) and ‘Akikiki (*Oreomystis bairdi*), are currently undergoing severe population declines [43, 44], suggesting that our results cannot be discounted. Moreover, it is possible that since many of our inputs were calculated from recently collected data, what we are witnessing in the models is a population already in decline.

Regardless of whether the Puaiohi population is stable or declining, it is precariously small, and therefore, highly susceptible to any of the threats it currently faces as well as stochastic events. Hence, it is important that the population is increased and that we strive to increase habitat quality to support greater numbers of this endemic bird. Our management scenario results indicate that a variety of real world, attainable management activities have the potential to increase Puaiohi numbers. Our models confirm our predictions that controlling predators, providing safer nesting alternatives, and supplementing food will increase Puaiohi population growth rate and size. Specifically, rat control, even at conservative levels, appeared to be the most effective method of increasing Puaiohi abundance, as did the provisioning of predator-proof nest boxes. Sensitivity analysis indicates that whichever management action is chosen should incorporate increasing female and juvenile survival.

### Caveats and future research

The mortality estimates used in our study are based on seven years of data, with somewhat limited re-sight data. It is possible that some emigration is being erroneously attributed to mortality, as it can be difficult to distinguish between the two in mark-recapture studies [45]. Given that our models are quite sensitive to changes in mortality estimates, and seem to be presenting a more pessimistic view than Puaiohi managers see in the field, further study to refine mortality estimates would be helpful.

In addition, reproductive estimates used in this study are based only on three years data and there can be variation in annual reproductive output, particularly associated with fluctuation in local weather patterns [14, 30]. Our dataset was not long enough to know whether these years were average background rate years, or if they represented the relatively rarer boom and bust years exhibited in very wet or very dry years, respectively. A longer study of annual reproductive output could help refine population analyses.

Further information on both current and future carrying capacity would also help improve model performance. At present, little is known regarding how many Puaiohi can be supported in their remaining habitat. Given the Puaiohi’s rather particular nest site preference for the ledges of narrow stream cuts, nest site availability may limit population size [46, 47]. Furthermore, daisy fleabane (*Erigeron annuus*), a relatively new invasive plant, grows well on steep rock walls, and may cover and eliminate formerly suitable nest sites [46], thereby decreasing an already limited resource. Other recent plant introductions, such as blackberry (*Rubus argutus*), Australian tree fern (*Cyathea cooperi*), and strawberry guava (*Psidium cattleianum*) have significantly altered areas currently and recently occupied by Puaiohi [11], and may change the Alakai’s future carrying capacity, as they have the potential to convert the forest canopy, understory and cliffs to novel habitats likely to be unsuitable to the species. At present, the Puaiohi’s native range is being taken over by kāhili ginger (*Hedychium gardnerianum*), a well-known invasive plant which blankets native forests and displaces native vegetation [48]. While ginger is a fruiting plant, unlike natives such as ʻolapa (*Cheirodendron trigynum*), lapalapa (*C*. *platyphyllum*), ʻōhiʻa ha (*Syzygium sandwicensis*) and kanawao (*Broussaisia argute*), its peak October-December fruiting period is not contemporaneous with the Puaiohi nesting period [49], and Puaiohi do not appear to eat it (Pejchar, unpubl data).

Inbreeding depression is another demographic aspect which we were unable to adequately model in this study, due to lack of data. We found that using default values in Vortex did not significantly affect our models, but it is unclear whether these default values are appropriate for this bird. Puaiohi, along with the rest of Kauai’s endangered birds, persist in numbers so low that lack of genetic diversity may pose potential problems, and population size may fall below the minimum viable population size recommended for long term maintenance of genetic diversity [6]. In fact, while the Puaiohi has been considered rare even historically [9], it does seem to have suffered a significant range contraction over the intervening years. Though the birds currently only occupy wet montane forest at 1,050 to 1,300 m [29], they have historically occupied lower mesic forests [9] and subfossil evidence of the species has been found in sinkholes and caves at sea level [50, 51]. Clearly, understanding the levels of genetic diversity still extant in the population could have ramifications for maintenance of the species. Furthermore, genetic studies could provide insight into the historical population size of the species. Recently, a captive breeding program for Puaiohi was discontinued due observed effects of inbreeding. Thus, managers are making important decisions based on a perceived loss of genetic diversity, and it would be useful to confirm the level left to be conserved in the wild population.

The effect of catastrophes on Puaiohi, particularly hurricanes and drought, is also unclear. Presumably, their preference for nesting in narrow stream corridors offers them some protection from the high winds associated with hurricanes [47]. Indeed, while 5 other species disappeared from the Alaka‘i following Hurricanes ‘Iwa (1982) and ‘Iniki (1992) [8], the Puaiohi persisted. However, the species was likely extirpated from at least two areas at the edges of its range following these two storms [47]. Furthermore, in at least one drought year, Puaiohi experienced exceptionally low reproductive output, likely due to limited food availability [14]. However, we only have data from a single drought year. Thus, while future hurricanes and droughts will likely affect the remaining Puaiohi population, their precise effects remain unclear, and as such, were not incorporated into our model. Further examination of how these catastrophes may affect Puaiohi populations is warranted.

Our lack of understanding of these processes may be further compounded by the unpredictability of future climate conditions, which will likely affect the long-term accuracy of our models’ predictions. Climate change has the potential to influence Puaiohi numbers in several ways. Hawai‘i tends to be drier and drought-prone during strong El Niño events and wet during La Niña events, although the effect of the latter is more variable [52, 53, 54]. This relationship between drought and El Niño is of concern as evidence suggests that El Niño and La Niña events have been more variable and intense over the past several decades, presumably due to the increase in environmental temperatures produced by anthropogenic climate change [55, 56, 57]. If this trend in variability and intensity persists, overall yearly precipitation may decrease across Hawai‘i [53], resulting in more years of low annual reproduction than Puaiohi can tolerate [30]. In addition, climate change scenarios often predict an increase in hurricanes [58]. Thus, understanding and modeling the effects of hurricanes on Puaiohi may become even more important to accurately predicting their future persistence.

Furthermore, because climate change may affect rates of malarial transmission, understanding the impacts of malaria on Puaiohi is urgently needed in order to evaluate the allocation of resources for decreasing the prevalence of malaria in Puaiohi habitat or to facilitate malarial resistance in the birds. The incidence of malaria is expected to increase with global climate change as warming temperatures allow the disease’s mosquito vector to penetrate into the current malaria transitional zone (altitudes >1,400m). Thus, mosquito vectors will likely expand their distribution to higher elevations, thereby increasing infection rates [59]. Furthermore, declines in high magnitude precipitation events which flush mosquito larvae from streams, may increase mosquito breeding habitat, and thus adult disease vectors [38] within the Puaiohi’s habitat.

While longer datasets and more support for some of our model assumptions would certainly increase our model confidence, it seems unlikely they would qualitatively affect our conclusions. Furthermore, the utility of PVA is less in the actual numbers it provides than as a means of comparing relative extinction risks, either between subpopulations of a species or between alternative management actions [18, 37, 60]. As with many forest bird species in Hawai‘i, gathering additional data is time consuming and expensive given the species’ often inaccessible locations and very small population sizes. Thus, while gaining more information would be useful, given the limited resources that can be dedicated to this species, we feel this study provides useful, actionable management recommendations with can help increase the Puaiohi population.

### Management recommendations

It seems clear that rat control within prime Puaiohi nesting habitat is critical for the species’ long-term survival. Previous research [14] demonstrated that intensive rat removal had significant positive impact on Puaiohi recruitment. Even at levels of impact below what Snetsinger achieved, rat control appears to be quite effective at increasing Puaiohi numbers (Fig 1). This has been shown to be true for the O‘ahu ‘Elepaio, [32, 33, 34]. Though rat control poses costs, technology exists to implement an effective rat control program. This effort might also act as an umbrella technique, whereby it would benefit not only the Puaiohi, but the other few remaining endemic birds of the Alaka‘i.

Providing predator-proof nest boxes also has potential to positively affect Puaiohi. This technique would not only increase female survival and reproductive output, but also increase the number of available nesting sites, a potential limiting factor for the species at present. Researchers have experimented with different nest box designs [61], but have yet to find a design readily accepted by Puaiohi. Research into effective nest box designs continues, however, providing some hope of an additional effective management option soon.

Other management activities exist that could increase Puaiohi populations and growth rates. Supplemental feeding, akin to the papaya and scramble eggs provided to recently released captive birds, may help increase carrying capacity and reproductive output [62, 63, 64, 65], though this is likely similar in its labor intensiveness to rat removal, with smaller pay offs. Removal of invasive plants to promote the return of native vegetation, and thereby, native food sources also has some potential for positive growth rates and number increases. In addition, this action would benefit many native plant species, as well as other endemic birds. Finally, steps can be taken to improve overall Puaiohi survival. One of the most likely means to do so would be the management of malaria. While Puaiohi do not seem to as susceptible to malaria as some Hawaiian endemic birds, evidence of the disease has been found in the species [31, 38, 39] and it likely does have an impact on overall species survival. Though more research is necessary to fully understand the impact of malaria on Puaiohi, removal of pigs may decrease to prevalence of suitable mosquito habitat, and thereby malaria, would likely be of some aid to sustaining populations. Pig removal would also limit the damage to native plants and the weed spread, thereby increasing habitat quality. It is also possible that factors outside those we have modeled may have an impact on Puaiohi. Future research should, in particular, investigate potential impacts of cats, owls and avian competitors. Because many of the issues facing Puaiohi are the same as those faced by the other Hawaiian forest birds, this, and all of the recommended management activities could have a substantial and valuable positive impact on the other few remaining endemic birds of the Alaka‘i.
